# Racial/Ethnic Differences in Vaping Product Use among Youth: A State-Level Analysis

**DOI:** 10.3390/ijerph20095729

**Published:** 2023-05-05

**Authors:** Christopher Cambron

**Affiliations:** College of Social Work, University of Utah, 395 South 1500 East, Salt Lake City, UT 84112, USA; chris.cambron@utah.edu

**Keywords:** e-cigarettes, racial/ethnic differences, youth substance use, health equity

## Abstract

National data suggest that non-Hispanic, White youth engage in electronic cigarette (e-cigarette) use at the highest rates. These results are not likely to be mirrored across regional contexts. State-representative data from Utah in 2019 (N = 58,689) were used to estimate the odds of lifetime and past 30-day vaping across seven racial/ethnic categories. Youth in grades 8, 10, and 12 (mean age 15.2; 52% female) self-reported race/ethnicity and vaping product use history, including e-cigarettes, vape pens, or mods. A Cox proportional hazards model estimated the cumulative probabilities for initiating vaping product use. The results indicated that American Indian or Alaskan Native, Black or African American, Hispanic/Latino, Multiracial, and Native Hawaiian or other Pacific Islander youth had significantly higher odds of both lifetime and past 30-day vaping compared to non-Hispanic White youth. The results showed significant variation in the cumulative probability of initiation by race/ethnicity, with Hispanic/Latino youth reporting the highest odds of initiation at each age. The regional patterns of vaping across racial/ethnic groups may not mirror national trends. State- and community-level data should be used to inform efforts to reduce e-cigarette use and promote health equity among youth.

## 1. Introduction

As of 2019, more than 35% of high school students in the United States (U.S.) had reported using an electronic cigarette (e-cigarette) at some point in their lifetime, and more than 20% reported use within the past month [1]. Given that e-cigarettes were first introduced in the U.S. in 2007, the rate of uptake for these devices among youth continues to be a source of substantial concern [2]. Health professionals have highlighted the potential negative effects of e-cigarettes on youth via early exposure to nicotine and other cancer-causing chemicals [2]. In December 2018, the Surgeon General declared e-cigarette use an epidemic among youth and recommended that communities take action to reduce youth e-cigarette use [3].

### 1.1. E-Cigarettes and Youth Health

The term e-cigarette describes a set of devices that share some common characteristics. These devices may look different and be referred to by a range of names, including e-cigs, cigalikes, vapes, vape pens, hookah pens, mods, tank systems, or electronic nicotine delivery systems [4]. Most e-cigarette devices contain four key components: a battery, a reservoir that contains a liquid, a heating element, and a mouthpiece. The heating element aerosolizes or vaporizes the liquid in the reservoir so that it can be inhaled by the user [2,4,5]. The liquids, also known as electronic liquids, e-liquids, e-juices, or vape juices, are available in multiple varieties and may consist of multiple different ingredients. These include a flavored or non-flavored liquid with no nicotine, a flavored or non-flavored liquid including nicotine, or a flavored or non-flavored substance that includes THC (tetrahydrocannabinol—the primary psychoactive compound in cannabis) or CBD (cannabidiol—a non-psychoactive compound in cannabis) [4]. Devices designed to vaporize THC, CBD, or any other psychoactive substances that do not contain nicotine do not meet the current legal definition of an e-cigarette as established by the U.S. Food and Drug Administration. They are classified as “vaping products”, of which an e-cigarette is one type. Additionally, a small proportion of e-liquids contain zero nicotine, but these devices are still considered e-cigarettes. This has led to some confusion among both researchers and the public. Importantly, the survey described in this study does not differentiate e-cigarettes and other vaping products. Youth were asked about vaping product use that included e-cigarettes, vape pens, and mods. As such, throughout the rest of this study, the phrase e-cigarette is used to indicate the wider class of vaping product use. Most e-liquids also include both Propylene Glycol (PG) and Vegetable Glycerin (VG) as preservatives. These are common food additives that may lead to lung inflammation when vaporized [5,6]. E-cigarettes may also contain diacetyl (a flavoring chemical), over 100 volatile organic compounds, heavy metals, and other ultrafine particles [4,6]. While the negative health effects of nicotine and exposure to heavy metals are well-established, scientists continue to assess the potential long-term consequences of exposure to other chemicals commonly found in e-cigarettes and other vaping products [7]. A lack of consistency across e-liquid brands, or even among products from the same brand, has made this task increasingly difficult for scientists.

Scientific understanding regarding the health effects of e-cigarette use has continued to evolve as e-cigarettes have become more prominent in the U.S. While it is generally believed that e-cigarettes are less harmful than traditional, combustible cigarettes, the recency of the e-cigarette epidemic among youth makes it difficult to assess the long-term health effects as of yet [8]. Additionally, the diversity of devices and liquid types that can be categorized as e-cigarettes increases the challenge of isolating the specific health effects associated with all or certain types of e-cigarettes. The following paragraph provides a brief overview of known health effects of common e-cigarette ingredients, including exposure to nicotine, heavy metals, THC, CBD, PG, VG, and other organic compounds.

First, the detrimental effects of nicotine exposure on youth are well-established [9]. In the short term, nicotine exposure can lead to nicotine dependence and the potential for severe withdrawal symptoms. In the longer term, nicotine exposure among children and adolescents primes the dopamine reward circuits in the brain for future addiction to nicotine, opioids, and other substances. Nicotine’s negative impacts on normal brain development have also been shown to manifest in increased behavioral problems, reduced self-control, and increased difficulty learning [5,9]. More generally, multiple studies suggest that nicotine exposure among children and youth negatively impacts the normal development of cardiovascular, respiratory, and immunological systems [9]. Many youths report a lack of awareness that e-liquids may contain nicotine [2]. Second, research suggests that as many as half of all e-liquids underreport their nicotine content by 10% or more [5]. Heavy metals have been identified in various types of e-liquids and devices. Given that many e-cigarette devices use a metal heating coil to vaporize e-liquid, the leaching of metals into the vapor may be common. A recent review found that the levels of heavy metals were higher in e-cigarette users compared to combustible cigarette smokers [10]. Heavy metals absorbed into the respiratory tract have many known serious health impacts, including decreased lung function, increased risk for cardiovascular and kidney disease, and links to multiple cancers. Heavy metals are a potent neurotoxin for youth that may do irreversible damage to cognitive development and functioning [10]. Third, THC and CBD are common ingredients in e-liquids vaped by youth despite being illegal in many states. As the levels of THC in cannabis have increased in recent decades, research suggests that THC exposure among youth can alter brain development, impair cognitive functioning, and increase the risk for psychiatric disorders over time [5]. Specifically regarding e-cigarettes, black market THC e-liquids appear to be largely responsible for the highly publicized cases of severe lung disease among youth in late 2019 and early 2020. The Centers for Disease Control and Prevention (CDC) has pinpointed Vitamin E acetate added to THC e-liquids as the main source of an outbreak of the lung disease commonly referred to as EVALI (e-cigarette or vaping product use-associated lung injury). Approximately 2800 cases of hospitalization and 68 deaths were attributed to EVALI, but the cases have substantially declined since early 2020 [11]. There is little information on the long-term effects of vaporized CBD, but cases of EVALI were reported from e-liquids thought to contain both CBD and Vitamin E acetate and no THC [12]. Fourth, PG and VG have been deemed safe for ingestion by the FDA, but there is little information on the long-term effects of inhaling vaporized PG and VG. Additionally, e-cigarette liquids and devices have been found to include numerous other substances, such as diacetyl (a flavoring chemical linked to lung disease) and over 100 volatile organic compounds. PG, VG, diacetyl, and other substances have not been approved for inhalation by the FDA, and little is known about their long-term effects [4]. Finally, nationally representative data suggest that youth using e-cigarettes are more likely to use combustible cigarettes in the future [13,14,15]. The dangers of combustible cigarettes across the lifetime are well known. Combustible cigarettes are estimated to cause more than 480,000 deaths per year and reduce life expectancy by at least 10 years [16].

### 1.2. Racial/Ethnic Disparities in E-Cigarette Use

Given the widespread adoption of e-cigarettes among youth and the numerous potential harms, the identification of youth experiencing elevated levels of exposure is necessary to guide prevention programming at national, state, and community levels. The extent to which racial/ethnic disparities in e-cigarette use may exist may serve to exacerbate already well-established health disparities among minority youth [17]. Data from nationally representative samples, including Monitoring the Future (MTF), Population Assessment of Tobacco and Health (PATH), and the National Youth Tobacco Survey (NYTS), have largely indicated that e-cigarettes may not be contributing to racial/ethnic health disparities. Research with these datasets has consistently reported that non-Hispanic, White youth and adults are using e-cigarettes at higher rates than non-Hispanic, Black, Hispanic/Latino, and other non-White youth [18,19,20,21]. Recent reviews, however, suggest that Hispanic/Latino youth may be experimenting with e-cigarettes at earlier ages compared to other racial/ethnic groups [17]. Importantly, few studies have compared e-cigarette use across multiple racial/ethnic groups, including American Indian or Alaskan Native (AI/AN), Native Hawaiian or other Pacific Islander (NH/PI), or Multiracial youth. State-level analyses of racial/ethnic disparities in youth e-cigarette use are largely unavailable. To add to this limited but growing body of literature, the current study examined lifetime, past 30-day, and age of initiation for e-cigarette use across seven racial/ethnic groups among a state-representative sample of youth from Utah in 2019. In comparison to other states, Utah youth use substances at lower rates [22]. The lower rates of substance use among Utah youth may reflect the influence of local religious norms and teachings associated with the Church of Jesus Christ of Latter-day Saints (LDS) that discourage alcohol, tobacco, and other drug use [22]. E-cigarettes have followed this trend with 30% of high school-age youth reporting lifetime e-cigarette use (compared to 35% in national estimates) and 14% reporting past 30-day use (compared to 20% in national estimates) in 2019 [1]. Given the well-known regional variation in multiple types of youth substance use [23], the results of the current study were expected to diverge from the national trends on e-cigarette use. These results can provide an important context for community public health practitioners working to prevent both youth e-cigarette use as well as racial/ethnicity-based health disparities in their states and communities.

## 2. Methods

### 2.1. Sample

The study draws on data from the Utah Department of Health (UDOH) Prevention Needs Assessment (PNA) survey from 2019. The PNA includes items from both the Youth Behavioral Risk Factor Surveillance System and the Communities that Care Youth Survey and is administered to a sample of Utah youth every two years via school districts by the Utah Division of Substance Abuse and Mental Health [24,25,26]. The PNA data are stratified by the school district, clustered by grade, and weighted to approximate the demographics of all Utah youth. The parents or guardians of the respondents were contacted by the school districts and provided active consent for participation. Approval for this study was provided by the Institutional Review Board of the UDOH (#0690). De-identified data from 8th, 10th, and 12th grade students that passed honesty and validation checks were used for the current study (N = 58,689). Weighted and unweighted sample characteristics are provided in Table 1.

### 2.2. Measures

Lifetime and past 30-day e-cigarette use. Lifetime and past 30-day e-cigarette use were each measured by a single question. The lifetime use item included the prompt “Have you ever used vape products such as e-cigarettes, vape pens, or mods?” with response options of 0 = No and 1 = Yes. The past 30-day use included the prompt “During the past 30 days, on how many days did you use vape products such as e-cigarettes, vape pens, or mods?” with seven response options (0 days, 1 or 2 days, 3 to 5 days, 6 to 9 days, 10 to 19 days, 20 to 29 days, All 30 days). The response categories indicating past 30-day e-cigarette use were collapsed such that 0 = No use and 1 = Any use. The measures of e-cigarette use employed by this study did not exclude youth also using combustible cigarettes. However, only 7.9% of youth reported lifetime dual combustible and e-cigarette use, and 1.2% of youth reported past 30-day dual combustible and e-cigarette use.

Age of e-cigarette initiation. The age of e-cigarette initiation was measured by a single item “If ever, how old were you when you first used a vape product (e-cigarettes, vape pens, or mods)?” with nine response options (Never, 10 or younger, 11, 12, 13, 14, 15, 16, 17 or older).

Demographics. Race/ethnicity was measured with a single item. Youth were provided with six response options and asked to select one or more categories. The response options provided were AI/AN, Asian, Black or African American (Black/AA), Hispanic/Latino, NH/PI, and White. Youth endorsing more than one racial/ethnic group were categorized as Multiracial. Youth categorized as AI/AN, Asian, Black/AA, NH/PI, and White all identified as non-Hispanic. Age and grade were each measured by a single item. Gender was measured by a single item with four response options (Woman/Girl, Man/Boy, Transgender, Other), and Transgender and Other were collapsed into a single category.

### 2.3. Analytic Plan

The data were analyzed with SPSS v27. Descriptive statistics and all the models were estimated using complex sample procedures to apply stratification by district, clustering by grade, and weighting to approximate population characteristics. Logistic regression models for lifetime and past 30-day e-cigarette use, including age and gender as covariates, were estimated with the CSLOGISTIC command. Odds ratios (OR) and 95% confidence intervals (CIs) were computed. Sensitivity tests examining racial/ethnic differences in lifetime and past 30-day e-cigarette use, while controlling for youth perceived risk of harm for e-cigarette use, produced substantively identical results to those presented below. A Cox proportional hazard model estimated the cumulative probability of e-cigarette initiation by race/ethnicity using the CSCOXREG command. The participants who never initiated e-cigarette use were right-censored at their current age. The Efron approximation was used to break ties and estimate the baseline survival function. The overall differences by race/ethnicity were evaluated via the Wald F statistic and showed significant variation [F(6, 87) = 64.75, *p* < 0.001]. As expected, the pattern of statistical significance for the results of the Cox model was substantively identical to the results of the logistic regression model examining lifetime e-cigarette use and, therefore, were not described in full. The proportional hazard assumption evaluated via the Wald F statistic did not indicate a violation [F(6, 82) = 1.23, *p* = 0.297]) [27]. A Cox proportional hazards model including gender as a covariate showed substantively identical results to those presented. Data were missing for less than 1.9% of the possible data points (7,502 out of 403,321) and were handled by listwise deletion.

## 3. Results

**Figure 1:**
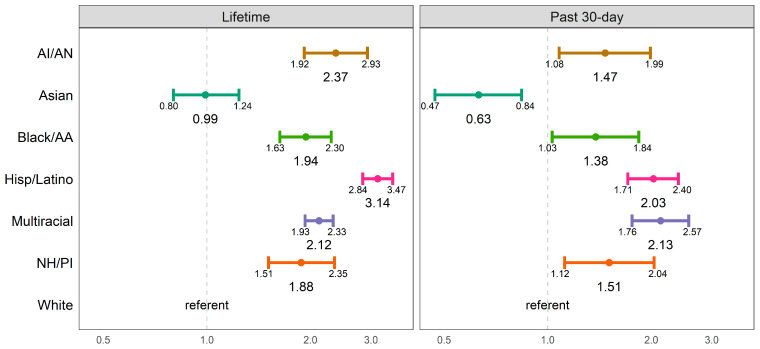
Odds ratios for lifetime and past 30-day e-cigarette use by race/ethnicity.

**Figure 2:**
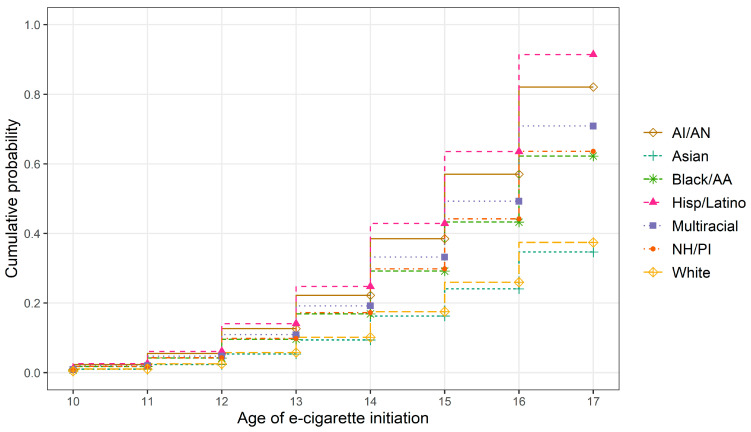
Cumulative probability of e-cigarette initiation by race/ethnicity and age.

Unweighted and weighted sample characteristics are provided in Table 1. The percentages of e-cigarette use across racial/ethnic groups by grade level are provided in Table 2. The results of the logistic regression models examining racial/ethnic differences in lifetime and past 30-day use are reported in Table 3 and Figure 1. The results indicated that youth reporting different racial/ethnic groups also reported significantly different rates of lifetime and past 30-day e-cigarette use. Compared to White youth, AI/AN (OR = 2.37, 95% CI [1.92, 2.93]), Black/AA (OR = 1.94, 95% CI [1.63, 2.30]), Hispanic/Latino (OR = 3.14, 95% CI [2.84, 3.47]), Multiracial (OR = 2.12, 95% CI [1.93, 2.33]), and NH/PI (OR = 1.88, 95% CI [1.51, 2.35]) youth had a significantly higher likelihood of lifetime e-cigarette use. Compared to White youth, AI/AN (OR = 1.47, 95% CI [1.08, 1.99]), Black/AA (OR = 1.38, 95% CI [1.03, 1.84]), Hispanic/Latino (OR = 2.03, 95% CI [1.71, 2.40]), Multiracial (OR = 2.13, 95% CI [1.76, 2.57]), and NH/PI (OR = 1.51, 95% CI [1.12, 2.04]) youth had a significantly higher likelihood of past 30-day e-cigarette use. Asian youth (OR = 0.63, 95% CI [0.47, 0.84]) had a significantly lower likelihood of past 30-day e-cigarette use compared to White youth. The cumulative probabilities from a Cox proportional hazards model for e-cigarette use initiation by race/ethnicity are reported in Table 4 and plotted in Figure 2. Hispanic/Latino youth showed the highest probability of e-cigarette use initiation at each age.

## 4. Discussion

The epidemic of e-cigarette use and potential harms to youth health continue to be a source of substantial concern among healthcare and public health professionals. The results of the current study indicate some local divergence from national e-cigarette use trends and suggest that regional variation may play an important role in understanding e-cigarette use across racial/ethnic groups. In Utah, Hispanic/Latino youth make up approximately 19% of the population of youth emphasizing the broad potential for harm from e-cigarette use. The already well-established health disparities among Hispanic/Latino and other minority youth in the United States [28] in conjunction with the numerous cancer-causing components of e-cigarettes highlight the need to raise awareness of regional variation in racial/ethnic disparities in e-cigarette use among youth.

The patterns of e-cigarette use by grade among Utah youth agree with emerging trends on e-cigarette use among younger Hispanic/Latinos. A recent review by Unger and Falcon stated “Hispanic adolescents are at risk for experimenting with e-cigarettes at early ages, potentially leading to nicotine dependence. However, for the older generations who were not exposed to e-cigarettes during adolescence, uptake of e-cigarettes is less prevalent among Hispanics than among some other groups” [17]. Recent analyses by Levy and colleagues also suggest that youth in Western states engage in lower levels of substance use overall [23]. Levy and colleagues note that “broad geographical regions group together disparate states and regions which may obscure more nuanced findings” regarding youth substance use [23]. The results of the current study echo these sentiments about regional variation in substance use but also note that some racial/ethnic groups may not follow the trend of lower substance use rates among youth in Western states. While nationally representative data on e-cigarette use among youth have largely revealed that non-Hispanic, White youth are engaging in e-cigarette use at the highest rates [18,19,20,21], the current study demonstrates that different patterns can emerge when considering regional data. This finding is not unsurprising when considering recent differences in the prevalence of youth cannabis use across state and national datasets. Recent analyses have suggested that large, state-representative samples of youth from Washington State were a more accurate gauge of cannabis use in those states when compared to findings from nationally representative datasets, such as Monitoring the Future [29]. Future studies should continue to compare national data sources to state and regional data sources to ensure that local communities and school districts have the most accurate data on current and emerging trends in youth substance use.

Limitations of the current study should be noted. Importantly, the results of the current study are largely descriptive and did not hypothesize about risk or protective factors that may help explain observed racial/ethnic differences in e-cigarette use. Recent research has noted that e-cigarette use among youth is linked to a range of individual, peer, parental, and socioeconomic factors [22,30,31]. In particular, studies have noted that youth engaging in combustible cigarette, alcohol, and cannabis use are highly susceptible to e-cigarette use [22]. Additionally, recent research suggests that the perception of risk for e-cigarette use may play a role in understanding racial/ethnic patterns of use across Hispanic/Latino and Black/African American dual e-cigarette/combustible cigarette users [32]. Future studies should explicitly be designed to understand the set of risk and protective factors that may help explain racial/ethnic disparities in e-cigarette use among youth across different regions and states. Second, some reports suggest that e-cigarette use, as well as multiple types of substance use, declined among youth across the United States in 2020 with the onset of the COVID-19 pandemic [29]. However, additional reports suggest that data on e-cigarette use collected via online platforms may systematically bias results [33]. It is yet to be determined if different groups of youth are more susceptible to online reporting biases related to e-cigarette use or if e-cigarette use among youth will rebound in the coming years. Third, data on self-reported e-cigarette use by youth also have the potential for underreporting regardless of the mode of survey administration. It is possible that some youth in this study have experimented with e-cigarette use but were unwilling to report it on the PNA survey. Given the religious norms against substance use among Utahans, [22] this possibility cannot be ruled out. Nonetheless, self-reported e-cigarette use in both national and other state-level data may be subject to similar regional or cultural biases. Future studies should explicitly seek to understand normative factors contributing to regional variation in youth substance use. Prospectively gathered longitudinal data are ideal for assessing the age of initiation for substance use [21]. Future studies assessing longitudinal cohorts of youth can provide additional insight into this important topic.

## 5. Conclusions

The results of the current study highlight the importance of examining regional or state-level differences in e-cigarette use among youth for both researchers and community practitioners [29]. Further research that explicitly compares states and regions can provide more information on these potential differences. Additionally, neither national nor state-level data, however, are able to offer information on the dynamic pathways through which e-cigarette use develops over time among youth. Longitudinal research examining daily and weekly e-cigarette use and other important dynamic risk and protective factors related to use are necessary. Studies specifically focusing on early experimentation with e-cigarettes among Hispanic/Latino youth can provide information regarding the potential for the early development of racial/ethnic disparities in e-cigarette use [17]. State- and community-level examination of these types of data can be used to inform prevention programs that are tailored to the highest-risk youth and promote health equity [17].

## Figures and Tables

**Table 1:** Sample characteristics and population estimates.

Variables		N	Unweighted M (SD), %	Weighted M (SD), %
Age		57,985	15.2 (1.6)	15.5 (1.7)
Grade				
	8th	25,581	43.6%	34.6%
	10th	20,376	34.7%	33.8%
	12th	12,732	21.7%	31.6%
Gender				
	Female	30,618	52.3%	51.2%
	Male	27,194	46.4%	48.2%
	Transgender	297	0.5%	0.2%
	Other	467	0.8%	0.4%
Race/ethnicity				
	American Indian/Alaskan Native	742	1.3%	0.8%
	Asian	1062	1.8%	1.8%
	Black/African American	794	1.4%	1.3%
	Hispanic/Latino	9241	15.8%	18.5%
	Multiracial	2813	4.8%	1.5%
	Native Hawaiian/Pacific Islander	734	1.3%	1.5%
	White	43,033	73.7%	74.4%
E-cigarette use				
	Lifetime	12,304	24.0%	26.0%
	Past 30-day	6489	11.5%	12.3%
	Age of initiation	12,189	14.2 (1.7)	14.4 (1.8)

Notes. Unweighted N = 58,589; M = mean; SD = standard deviation; weighted %’s approximate population characteristics.

**Table 2:** Lifetime and past 30-day e-cigarette use by race/ethnicity in Utah and national data from Monitoring the Future.

	8th		10th		12th		Total	
Race/Ethnicity	Lifetime	Past 30-Day	Lifetime	Past 30-Day	Lifetime	Past 30-Day	Lifetime	Past 30-Day
AI/AN	23.4%	11.6%	29.1%	16.6%	42.1%	16.2%	30.2%	14.3%
Asian	12.1%	6.2%	20.5%	8.8%	21.6%	5.8%	18.0%	7.0%
Black/AA	19.3%	8.7%	27.8%	17.1%	38.6%	16.6%	27.4%	13.7%
Hispanic/Latino	31.6%	16.8%	39.9%	19.0%	48.4%	21.4%	39.4%	18.9%
Multiracial	22.4%	11.6%	34.2%	21.9%	44.0%	26.0%	33.2%	19.6%
NH/PI	17.7%	8.2%	33.6%	19.6%	35.8%	19.0%	28.7%	15.4%
White	10.6%	5.3%	21.4%	12.1%	27.3%	14.6%	19.7%	10.6%
Utah (all youth)	15.2%	7.8%	25.3%	13.6%	31.5%	15.9%	26.0%	12.3%
National (all youth) ^1^	20.3%	12.2%	36.3%	25.0%	40.8%	30.9%	36.7%	22.5%

Note. %’s weighted to approximate population characteristics; American Indian or Alaskan Native (AI/AN), Black or African American (Black/AA), Native Hawaiian or other Pacific Islander (NH/PI); 1 = national data from Monitoring the Future [1].

**Table 3:** Results of logistic regression models for lifetime and past 30-day e-cigarette use by race/ethnicity.

	Lifetime ^a^				Past 30-Day ^b^			
Race/Ethnicity	Est.	SE	*p*	OR (95% CI)	Est.	SE	*p*	OR (95% CI)
AI/AN	0.863	0.107	<0.001	2.37 (1.92, 2.93)	0.382	0.153	0.015	1.47 (1.08, 1.99)
Asian	−0.008	0.111	0.940	0.99 (0.80, 1.24)	−0.463	0.145	0.002	0.63 (0.47, 0.84)
Black/AA	0.661	0.087	<0.001	1.94 (1.63, 2.30)	0.320	0.145	0.030	1.38 (1.03, 1.84)
Hispanic/Latino	1.144	0.050	<0.001	3.14 (2.84, 3.47)	0.706	0.085	<0.001	2.03 (1.71, 2.40)
Multiracial	0.752	0.048	<0.001	2.12 (1.93, 2.33)	0.754	0.096	<0.001	2.13 (1.76, 2.57)
NH/PI	0.632	0.111	<0.001	1.88 (1.51, 2.35)	0.412	0.152	0.008	1.51 (1.12, 2.04)
White	referent			1 (1, 1)	referent			1 (1, 1)
Intercept	−5.130	0.686	<0.001	-	−5.113	0.662	<0.001	-

Note. a = unweighted N = 55,341; b = unweighted N = 55,559; Est. = estimate; SE = standard error; *p* = *p*-value; OR = odds ratio; CI = confidence interval; models stratified by school district, clustered by grade, and weighted to approximate population characteristics; models included age and gender as covariates; American Indian or Alaskan Native (AI/AN), Black or African American (Black/AA), Native Hawaiian or other Pacific Islander (NH/PI).

**Table 4:** Cumulative probability of e-cigarette initiation by race/ethnicity and age.

	Race/Ethnicity						
Age	AI/AN	Asian	Black/AA	Hisp/Latino	Multiracial	NH/PI	White
10	0.010	0.004	0.008	0.011	0.009	0.008	0.005
11	0.023	0.010	0.018	0.026	0.020	0.018	0.011
12	0.055	0.023	0.041	0.061	0.047	0.042	0.025
13	0.126	0.053	0.096	0.140	0.109	0.098	0.057
14	0.222	0.094	0.169	0.248	0.192	0.172	0.101
15	0.385	0.162	0.292	0.429	0.332	0.298	0.175
16	0.570	0.241	0.432	0.635	0.492	0.442	0.260
17	0.821	0.346	0.622	0.914	0.709	0.636	0.374

Note. unweighted N = 56,593; Wald test for differences in hazard rates F(6, 87) = 64.75, *p* < 0.001; models stratified by school district, clustered by grade, weighted to approximate population characteristics; American Indian or Alaskan Native (AI/AN), Black or African American (Black/AA), Hispanic/Latino (Hisp/Latino), Native Hawaiian or other Pacific Islander (NH/PI).

## Data Availability

De-identified data are available by request from the Utah Department of Health.
